# Do students’ attitudes toward required readings and service-learning for a Medical Humanities course predict their perception of whether the course fosters their personal and professional development?

**DOI:** 10.3389/fmed.2025.1636277

**Published:** 2025-07-25

**Authors:** Shane L. Rogers, Lon J. Van Winkle

**Affiliations:** ^1^School of Arts and Humanities, Edith Cowan University, Joondalup, WA, Australia; ^2^Master of Science in Biomedical Sciences (MSBS) Program, Rocky Vista University, Englewood, CO, United States; ^3^Department of Biochemistry, Midwestern University, Downers Grove, IL, United States

**Keywords:** team-based learning, service-learning, professional development, implicit biases, listening, compassion, critical reflection, empathy

## Abstract

**Purpose:**

To determine whether prospective medical students’ attitudes toward readings and service-learning for a Medical Humanities course predict their perceived impact of the course on their critical reflection and empathy for their peers.

**Methods:**

Following a Medical Humanities course, students completed surveys concerning their attitudes toward team-based service-learning and readings required for the course. And they completed surveys designed to measure their empathy for one another and thoughts and feelings about the course (e.g., “owing to this course, I have changed the way I look at myself” = critical reflection).

**Results:**

Students’ positive attitudes toward service-learning and readings for the course correlated positively with each other and with their critical reflection (CR) and empathy for their peers (*r* = 0.28 to 0.63, *p* < 0.05 to 0.0001).

**Conclusion:**

Positive attitudes toward team-based service-learning predicted students’ perceptions of the Humanities course’s impact on both critical reflection and peer empathy, whereas attitudes toward required readings played a significantly lesser role.

## Introduction

Empathy is a foundational element of high-quality healthcare, associated with better patient outcomes, improved satisfaction, and greater provider wellbeing (1). Yet many healthcare professionals struggle to maintain empathy under pressure with nearly half experiencing “compassion fatigue” (2, 3). As a result, medical students often see supervisors whose actions do not consistently model empathy and compassion towards both patients and colleagues (4). While structured empathy training could address these challenges (1, 5–7), efforts to embed such training across healthcare curricula remain uneven and fragmented. This raises a key question: Why are healthcare education and refresher programs still slow to integrate Medical Humanities as a core requirement, despite its strong evidence base for fostering empathy and reflection?

One reason may be uncertainty around what aspects of Medical Humanities training are most impactful. Both teams-based service learning and reflective engagement with readings are commonly used components, but they differ in focus and demands. Service-learning emphasizes active engagement with communities, often confronting students with real-world dissonance that can trigger reflection and perspective-taking (8–11). When paired with written critical reflection, such experiences have been shown to reduce unconscious biases, strengthen empathy, and, thus, eventually improve the quality of patient care (12–18). These gains, however, may fade if reflection is not ongoing (19–22).

In contrast, reading and discussing humanities texts may foster empathy through exposure to others’ lived experiences and structured class dialogue, without requiring the logistical effort of community-based placements. But few studies have directly compared the influence of these two components within the same course or student population.

In our previous studies, we looked at whether improved empathy in aspiring medical students depended on taking part in service-learning, or if simply writing about the challenges of medical training, clinical practice, and maintaining humanism would be enough to foster higher empathy and compassion (11). We found that students’ Reflective Capacity (23) and Jefferson Scale of Empathy (24) scores increased when a Medical Humanities course required them to perform team service-learning and write critical reflections on those experiences (treatment group). Students who did not engage in service-learning (control group) did not experience these gains.

However, in our prior research (11), there were other important differences between our treatment and control groups. Specifically, our experimental and control groups were divided by whether the students attended school at our Colorado or Utah campuses. This meant variations in campus environments and personal factors influenced each student’s choice of location. In addition, the details of students’ reflection assignments differed because the focus of the assignments in Colorado were on service-learning while the Utah group focused more on reflection assignments tied to readings for their Humanities course. Moreover, there were differences in the reading assignments in Colorado and Utah, and the impact of the readings on students’ empathy and professional development have not been examined.

Due to these limitations in the between-groups design of our prior research (25, 26), in the present study we sought to re-examine the impact of required service-learning and readings for the Medical Humanities course on students’ empathy, professional development, and ability to perform critical reflection in a single population of prospective medical students (i.e., within-participants design). Building on our earlier findings that service-learning and reflective activities can enhance empathy and critical reflection (11, 16, 19, 20), we propose several hypotheses regarding the influence of both service-learning and required readings in a Medical Humanities course. We anticipate that a revised survey, which asks students to consider statements such as “owing to this course, I have changed the way I look at myself,” will prove to be a reliable and valid measure of critical reflection (CR).

We tested six hypotheses:

1. Students who report more positive attitudes toward the service-learning project will also report higher perceived impact of the course and a greater ability to perform critical reflection.
2. Favorable attitudes toward service-learning will be associated with greater empathy among peers.
3. Students’ attitudes toward required readings will predict their sense of the course’s impact and their level of critical reflection.
4. Students’ attitudes toward required readings will predict the empathy they feel for one another.
5. Students’ attitudes toward service-learning will more strongly predict their perceived impact of the course (i.e., on CR) than attitudes toward the required readings.
6. Students’ attitudes toward service-learning will more strongly predict their perceived impact of the course on empathy for peers than attitudes toward the required readings.

## Methods

### Participants and procedure

Fifty participants matriculated as first-year Master of Science in Biomedical Sciences (MSBS) students at Rocky Vista University-Colorado campus in August of 2024. Of such students, 92% graduate from the MSBS program and are admitted usually to an osteopathic school of medicine (27).

As part of the MSBS program, students enrolled in a Medical Humanities course, which required them to engage with selected humanities texts and participate in team service-learning projects. On the first day of class, students were randomly assigned to teams of six or seven. Each team met regularly throughout the semester to select and complete a community service project, with final approval provided by the course director (LJV). The list of all the service-learning projects chosen were: Alzheimer’s Association (bake sale fundraiser), Big Brothers Big Sisters, DAWN clinic (in partnership with CU Anschutz), Denver Botanic Gardens, Denver Rescue Mission, Joy’s Kitchen, Orchard Park health Care Center, Project C.U.R.E., Rocky Vista University Clinic, SECOR Cares, Special Olympics, Susan G. Komen Breast Cancer Awareness Marathon, Toys for Tots, Walk to End Alzheimer’s.

Each student completed a minimum of 5 h of service, and teams submitted written critical reflections four times over the semester, with one submission each month. These reflections addressed both their service-learning experiences and other aspects of the MSBS program, including the required readings. Our operational definition of critical reflection (CR), published previously (16), involves students recognizing when their thoughts or behaviors misalign with their personal or humanistic values, experiencing internal dissonance, and beginning to reconcile these inconsistencies in order to act more intentionally and empathetically.

In parallel, students engaged in weekly reading and discussion of four selected texts (28–31):

- *The Compassionate Connection: The Healing Power of Empathy and Mindful Listening* by David Rakel
- *The People’s Hospital: Hope and Peril in American Medicine* by Ricardo Nuila
- *What Patient’s Say, What Doctors Hear* by Danielle Ofri
- *Legacy: A Black Physician Recons with Racism in Medicine* by Uché Blackstock

Students read selections from one of the texts before each class meeting and a quiz was used to help foster group discussion. For example, for the text ‘The Compassionate Connection’, a couple of example quiz questions were: “Adverse childhood experiences are associated with increased frequency of which of the following when these children become adults?” (A. Cardiovascular disease, B. Cancer, C. Chronic lung disease, D. Liver disease, E. All the answers) and “Which of the following pairs of characteristics in healthcare providers are most concordant in fostering the best outcomes of patient care?” (A. Empathy and empathic distress, B. Empathy and compassion, C. Compassion and empathic distress, D. All the pairs are equally concordant, E. All the pairs are equally discordant). Class periods (50 min) emphasized small-group and whole-class discussion of the readings, with a focus on fostering empathy, critical reflection, effective communication and compassion, including toward one another as peers. For any reader interested in more information on the service learning and reading activities that are conducted as part of our Medical Humanities course please contact the corresponding author.

This structure provided two distinct yet interrelated pathways for developing empathy and reflective capacity:

1. Service-learning: Which exposed students to real-world contexts and required them to reflect on lived experiences.
2. Humanities readings: Which exposed students to diverse perspectives though narrative and analytical discussions.

The current study examined the degree to which students’ attitudes towards each of these components predicted perceived personal and professional development outcomes, including empathy for peers and critical reflection. The surveys used to obtain measures for these variables of interest are detailed below.

### Surveys and experimental design

Paper versions of four surveys were completed by students on the final day of the Medical Humanities course. Surveys were distributed randomly to students in numbered packets of four, so that survey responses by each student could be paired with one another. The packets could not, however, be associated with individual students, and they were completed anonymously and voluntarily. One of us (LV) collected the paper surveys and processed them for data analysis.

The surveys included the CR survey (Supplementary material A), which was modified from an original “Reflection Questionnaire” (32), the survey on readings (Supplementary material B), our questionnaire on students’ attitudes toward service learning (SASL) (33), and a survey on students’ empathy for their peers (27). The eight-item SASL survey (range of possible scores = 1 to 7) and the 12-item empathy survey (range of possible scores = 0 to 4) were validated and found to be reliable in prior studies (27, 33). Here we report the reliability and test the validity of the CR survey and our readings questionnaire.

The CR survey was adapted from a “Reflection Questionnaire.” We acknowledge that the copyright of this Questionnaire is owned by the authors to satisfy their conditions for its use (32). It was designed originally to measure Habitual Action, Understanding, Reflection, and Critical Reflection (CR). Of the 12 items in this original questionnaire, we adapted the four items measuring CR for use in our study (Supplementary material A). In our adaptation, we used a seven-point Likert scale from “Strongly Disagree” [1] to “Strongly Agree” [7]. Thus, possible survey scores ranged from one to seven depending on the average responses of students to the four items. Items were of the form “As a result of this course, I have changed the way I look at myself” (item 1 in Supplementary material A).

The readings questionnaire was designed to measure the extent to which students felt that each of 12 survey items fostered their “ability to show compassion and listen in more profound ways” (Supplementary material B). Items included reference not only to reading assignments in each of the four books themselves, but also to discussions of the readings in their teams of six or seven students and by the whole class of 50 students. Students were instructed to use a seven-point Likert scale from “Strongly Disagree” [1] to “Strongly Agree” [7] to rate each item. So possible scores ranged from one to seven depending on the average responses of students to the 12 items. Items were of the form “Readings in the book *The Compassionate Connection: The Healing Power of Empathy and Mindful Listening* by David Rakel” fostered my ability to show compassion and listen in more profound ways (item 1 in Supplementary material B).

The Rocky Vista University Institutional Review Board (IRB) found that this study (IRB #2025–029) satisfies the criteria for exemption.

### Statistical analyses

GraphPad Prism 10.4.1 Software Inc. (La Jolla, CA) was used to calculate survey mean, standard deviation, standard error of the mean, and Cronbach’s alpha values. This software was also used to calculate correlation (*r*) values among students’ various survey scores. Multiple regression analyses were conducted using the Statistical Package for the Social Sciences (SPSS).

## Results

Descriptive statistics for each questionnaire are shown in Table 1. The CR survey, which asks students to consider statements such as “owing to this course, I have changed the way I look at myself,” proved to be a reliable and valid measure of critical reflection (CR). Cronbach’s alpha value for the CR survey was 0.76, thus establishing its reliability. And its validity is shown by survey results that are predicted by students’ attitudes toward required service-learning (SASL) and readings for a Medical Humanities course, as described and discussed below (Table 2).

**Table 1:** Survey values: descriptive statistics.

Survey	SASL	Readings	Empathy	CR
Mean	5.83	5.99	3.00	5.28
Std. deviation	0.706	0.765	0.646	0.946
Std. error	0.100	0.108	0.091	0.134
*N*	50	50	50	50

**Table 2:** Correlation (*r*) values among the four surveys used in this study.

	SASL	Readings	Empathy	CR
SASL	–			
Readings	0.54*	–		
Empathy	0.52*	0.40*	–	
CR	0.63*	0.28*	0.33*	–

SASL = Students’ attitudes toward required service-learning, and CR = Critical reflection. *p < 0.05.

The reliability and validity of the readings survey has also not been assessed previously. Here we found the survey to have a Cronbach’s alpha value of 0.86, thus supporting the conclusion that it is a reliable measure of students’ attitudes toward the readings. And its validity is shown by reading survey results that are associated with students’ CR and empathy (Table 2). Moreover, every student’s overall opinion of the readings was favorable (i.e., score above 4.0).

*Hypothesis 1*: Students who report more positive attitudes toward the service-learning project will also report higher perceived impact of the course and a greater ability to perform CR.

In our prior studies, we showed the SASL to be a reliable and valid measure of students’ attitudes toward performing team-based service-learning (33). Here, we found the Cronbach’s alpha value for SASL to be 0.80 for the present cohort of students. And every student’s overall SASL score was favorable (i.e., above 4.0). Finally, students’ scores on SASL were highly correlated with their CR scores (*r* = 0.63, Table 2), thus serving to verify Hypothesis 1.

*Hypothesis 2*: Favorable attitudes toward service-learning will be associated with greater empathy among peers.

In another study, we found the empathy survey to be a reliable and valid measure of students’ empathy for one another (27). For the present cohort of students, the empathy survey had a Cronbach’s alpha value of 0.89. Moreover, students’ SASL and empathy scores were highly correlated (*r* = 0.52, Table 2) also verifying Hypothesis 2.

*Hypothesis 3*: Students’ attitudes toward required readings will predict their sense of the course’s impact and their level of CR.

Both the readings survey and the CR questionnaire were found to be reliable measures of the pertinent students’ attitudes. (See above.) Students’ scores for their attitudes toward these readings were correlated with their CR scores (0.28, Table 2), thus supporting Hypothesis 3.

*Hypothesis 4*: Students’ attitudes toward required readings will predict the empathy they feel for one another.

Students’ empathy scores were moderately correlated with their scores on attitudes toward required readings (0.40, Table 2), which supports Hypothesis 4.

*Hypothesis 5*: Students’ attitudes toward service-learning will more strongly predict their perceived impact of the course (i.e., on CR) than attitudes toward the required readings.

A multiple regression analysis was conducted to predict student perceived impact of the course on their critical reflection ability from their self-reported attitude toward service-learning (SASL) and appraisal of readings (READINGS). Together the predictors accounted for 40% of the variance in their perceived impact of the course on critical reflection [*R^2^* = 0.40, *F*(2,47) = 15.69, *p* < 0.001]. Only SASL (standardized beta = 0.67, *p* < 0.001) remained a significant predictor while READINGS did not (standardized beta = −0.08, *p* = 0.55). Results suggest that self-reported attitude towards service-learning is a more important predictor of student appraisal of course impact on their critical reflection than attitude towards reflective reading.

*Hypothesis 6*: Students’ attitudes toward service-learning will more strongly predict their perceived impact of the course on empathy for peers than attitudes toward the required readings.

A multiple regression analysis was conducted to predict student self-reported empathy towards peers from their self-reported attitude toward service-learning (SASL) and appraisal of readings. Together the predictors accounted for 29% of the variance in their self-reported empathy towards peers [*R^2^* = 0.29, *F*(2,47) = 9.70, *p* < 0.001]. Once again, only SASL (standardized beta = 0.43, *p* < 0.001) remained a significant predictor while READINGS did not (standardized beta = 0.17, *p* = 0.25). Results suggest that self-reported attitude towards service-learning is a more important predictor of student self-reported empathy towards peers than attitude towards reflective reading.

## Discussion

Our findings support all six hypotheses and align with conclusions from earlier research (11, 16, 19, 20). When service-learning is incorporated into a Medical Humanities course, students report both professional growth and higher empathy (11). In the current study, students’ attitudes toward service-learning were positively associated with both their perception of how well the course fostered critical reflection (CR) and their empathy toward peers. Although CR and empathy were moderately correlated (*r* = 0.33, Table 2), this does not necessarily imply a direct cause-and-effect relationship.

In a prior study, we found that reflective capacity (RC) correlated with Jefferson Scale of Empathy scores (11), and experiences in the Medical Humanities course led to significant increases in both measures. However, there was no significant correlation between changes in RC and changes in empathy (11). Likewise, in the present study, the modest correlation between CR and empathy (*r* = 0.33) disappeared (*r* = 0.01) once attitudes toward service-learning (SASL) and reading scores were included in a multiple regression analysis.

Regression analyses on the data also support the conclusion that students’ positive attitudes towards service-learning offer a much stronger explanation of both their critical-reflection growth and empathy than their appraisal of reflective readings. In other words, although reflective reading may be valuable, students’ enthusiasm for and engagement in service-learning activities seems to be the key factor that shapes their perceptions of how much the course impacts their reflective abilities and empathy.

Importantly, this study used a within-participants design, avoiding some confounds that limited our previous research. All students completed the same course with the same structure, allowing us to directly compare the relative influence of two key components (service learning and reflective readings) within the same cohort. The design provides clearer evidence for the utility of both components, with a slight advantage for the service-learning component.

These results align with earlier studies comparing control and experimental groups, where only the experimental group participated in service-learning (11). While randomized controlled trials are ideal in principle, the main ethical concern is that some students might be denied a beneficial educational intervention. Given the existing body of evidence suggesting that reflective exercises and service-learning promote professional development and empathy (11–17, 19–22), withholding such activities from a control group could disadvantage certain students, potentially affecting their professional growth and patient care outcomes. Even a single 40-min empathy workshop has been shown to boost Jefferson Scale of Empathy scores in students, although the effect may be temporary (34). The central challenge is to maintain healthcare professionals’ compassion for patients and empathy for one another throughout their careers. Numerous programs have been developed to support this goal, and the present findings add to a growing body of research indicating that structured, longitudinal engagement through Medical Humanities courses may provide a durable foundation for empathy and reflective practice (35).

For example, a 6-week Medical Humanities module, which increased students’ Jefferson Scale of Empathy scores after only 16 h of instruction (36), and narrative medicine education, which includes extensive reflective writing, also improved academic performance and empathy among female nursing students, with gains persisting for at least two and a half years (37). However, other studies suggest that without a reflective writing component, such benefits may vary, particularly by gender or training stage (38). Our findings reinforce the view that structured service-learning, paired with critical reflection, is a key mechanism for fostering durable professional growth.

Considering all the findings discussed here, one pressing question remains: Why are healthcare training programs and refresher courses still slow to integrate Medical Humanities as a core requirement? A growing body of research shows that sustained engagement with humanities content, especially when paired with critical reflection and service-learning, consistently boosts learners’ empathy (1, 11, 16, 19, 20, 35, 37). Higher empathy, in turn, is linked to better patient outcomes (39–41), greater patient satisfaction (42, 43), and improved practitioner wellbeing (44–47).

Beyond these clinical benefits, Medical Humanities courses prompt students to recognize and challenge the hidden assumptions and structural biases that shape healthcare delivery (11, 16, 19, 20, 35). By embedding empathy and reflection into routine professional thinking, these courses help foster a culture of social justice and equitable care (48–52). Although resistance to “soft-science” content still exists in some programs (53), our results suggest that when Medical Humanities is integrated thoughtfully into the curriculum, students view it not as peripheral, but as central to becoming competent, compassionate professionals. In short, Medical Humanities is more than an academic add-on; it is a proven pathway to empathetic clinicians, healthier patients, and a more just healthcare system.

### Limitations

This study was conducted with a small population of 50 premedical students at a single institution, which limits generalizability. In addition, our use of correlational data limits the ability to infer causality. However, the within-subjects design helps control for contextual and demographic confounds, and the findings are consistent with prior research (11, 16, 19, 20). Moreover, they align with broader evidence showing that Medical Humanities Programs foster development of empathy and compassion in healthcare professional students and providers (35).

## Conclusion

Positive attitudes toward team-based service-learning predicted students’ perceptions of the Humanities course’s impact on both critical reflection and peer empathy, whereas attitudes toward required readings played a significantly lesser role. These findings suggest that structured service-learning may be a particularly powerful and scalable lever for embedding Medical Humanities more effectively into healthcare training. When thoughtfully implemented, it has the potential to shape not only what students know, but also how they see themselves and others in the practice of care.

## Data Availability

The original contributions presented in the study are included in the article/Supplementary material, further inquiries can be directed to the corresponding author.
