# Supplementary material for: Do students’ attitudes toward required readings and service-learning for a Medical Humanities course predict their perception of whether the course fosters their personal and professional development?
Source: Front Med (Lausanne). 2025 Jul 25;12:1636277. doi: 10.3389/fmed.2025.1636277 (PMC12331726; doi:10.3389/fmed.2025.1636277)
Supplement: Supplementary file 1 [file Data_Sheet_1.pdf]

Supplementary Material A

Survey Regarding Your Actions and Thinking in Medical Humanities

A. Please use the scale below to indicate the extent to which you agree with each statement about your actions and thinking in Medical Humanities.

1 **Strongly Disagree**	2 **Disagree**	3 **Somewhat Disagree**	4 **Neither Agree/ Disagree**	5 **Somewhat Agree**	6 **Agree**	7 **Strongly Agree**

1. As a result of this course, I have changed the way I look at myself. _____
2. This course has challenged some of my firmly held ideas. _____
3. As a result of this course, I have changed my normal way of doing things. _____
4. During this course I discovered faults in what I had previously believed to be right. _____

B. Please comment on any or all the items above.

Supplementary Material B

Survey Regarding Readings and Discussions in Medical Humanities

A. Please use the scale below to indicate the extent to which you think and feel that each of the following fostered your ability to show compassion and listen in more profound ways.

1<br>**Strongly Disagree**	2<br>**Disagree**	3<br>**Somewhat Disagree**	4<br>**Neither Agree/ Disagree**	5<br>**Somewhat Agree**	6<br>**Agree**	7<br>**Strongly Agree**

1. Readings in the book "The Compassionate Connection: The Healing Power of Empathy and Mindful Listening" by David Rakel. _____
2. Team discussions about the book "The Compassionate Connection: The Healing Power of Empathy and Mindful Listening" _____
3. Discussions with the whole class about the book "The Compassionate Connection: The Healing Power of Empathy and Mindful Listening" _____
4. Readings in the book "What Patients Say, What Doctors Hear" by Danielle Ofri. _____
5. Team discussions about the book "What Patients Say, What Doctors Hear" _____
6. Discussions with the whole class about the book "What Patients Say, What Doctors Hear" _____
7. Readings in the book "The People's Hospital: Hope and Peril in American Medicine" by Ricardo Nuila. _____
8. Team discussions about the book "The People's Hospital: Hope and Peril in American Medicine" _____
9. Discussions with the whole class about the book "The People's Hospital: Hope and Peril in American Medicine" _____
10. Readings in the book "Legacy: A Black Physician Recons with Racism in Medicine" by Uché Blackstock. _____
11. Team discussions about the book "Legacy: A Black Physician Recons with Racism in Medicine" _____
12. Discussions with the whole class about the book "Legacy: A Black Physician Recons with Racism in Medicine" _____

B. Please comment on any or all the items above.
